# Effects of the New Generation Synthetic Reconstituted Surfactant CHF5633 on Pro- and Anti-Inflammatory Cytokine Expression in Native and LPS-Stimulated Adult CD14^+^ Monocytes

**DOI:** 10.1371/journal.pone.0146898

**Published:** 2016-01-20

**Authors:** Kirsten Glaser, Markus Fehrholz, Tore Curstedt, Steffen Kunzmann, Christian P. Speer

**Affiliations:** 1 University Children´s Hospital, University of Würzburg, Würzburg, Germany; 2 Department of Molecular Medicine and Surgery, Karolinska Institutet at Karolinska University Hospital, Stockholm, Sweden; Centre Hospitalier Universitaire Vaudois, FRANCE

## Abstract

**Background:**

Surfactant replacement therapy is the standard of care for the prevention and treatment of neonatal respiratory distress syndrome. New generation synthetic surfactants represent a promising alternative to animal-derived surfactants. CHF5633, a new generation reconstituted synthetic surfactant containing SP-B and SP-C analogs and two synthetic phospholipids has demonstrated biophysical effectiveness *in vitro* and *in vivo*. While several surfactant preparations have previously been ascribed immunomodulatory capacities, *in vitro* data on immunomodulation by CHF5633 are limited, so far. Our study aimed to investigate pro- and anti-inflammatory effects of CHF5633 on native and LPS-stimulated human adult monocytes.

**Methods:**

Highly purified adult CD14^+^ cells, either native or simultaneously stimulated with LPS, were exposed to CHF5633, its components, or poractant alfa (Curosurf^®^). Subsequent expression of TNF-α, IL-1β, IL-8 and IL-10 mRNA was quantified by real-time quantitative PCR, corresponding intracellular cytokine synthesis was analyzed by flow cytometry. Potential effects on TLR2 and TLR4 mRNA and protein expression were monitored by qPCR and flow cytometry.

**Results:**

Neither CHF5633 nor any of its components induced inflammation or apoptosis in native adult CD14^+^ monocytes. Moreover, LPS-induced pro-inflammatory responses were not aggravated by simultaneous exposure of monocytes to CHF5633 or its components. In LPS-stimulated monocytes, exposure to CHF5633 led to a significant decrease in TNF-α mRNA (0.57 ± 0.23-fold, p = 0.043 at 4h; 0.56 ± 0.27-fold, p = 0.042 at 14h). Reduction of LPS-induced IL-1β mRNA expression was not significant (0.73 ± 0.16, p = 0.17 at 4h). LPS-induced IL-8 and IL-10 mRNA and protein expression were unaffected by CHF5633. For all cytokines, the observed CHF5633 effects paralleled a Curosurf®-induced modulation of cytokine response. TLR2 and TLR4 mRNA and protein expression were not affected by CHF5633 and Curosurf®, neither in native nor in LPS-stimulated adult monocytes.

**Conclusion:**

The new generation reconstituted synthetic surfactant CHF5633 was tested for potential immunomodulation on native and LPS-activated adult human monocytes. Our data confirm that CHF5633 does not exert unintended pro-inflammatory effects in both settings. On the contrary, CHF5633 significantly suppressed TNF-α mRNA expression in LPS-stimulated adult monocytes, indicating potential anti-inflammatory effects.

## Introduction

Surfactant replacement therapy using exogenous surfactant preparations derived from bovine or porcine lungs has significantly improved the outcome in respiratory distress syndrome (RDS) in preterm infants and has become the standard of care for the prevention and treatment of RDS [1–8]. Pulmonary surfactant prevents alveolar collapse and improves lung compliance by significant reduction of surface tension at low lung volumes or minimal alveolar size–both conditions particulary prone to collapse according to Laplace´s tidal equations [9–11]. It promotes gas exchange, thus, allowing for rapid de-escalation of ventilation strategy and the use of lower concentrations of oxygen [12]. A number of animal-derived surfactants are available, isolated mainly from porcine or bovine lungs [6,8]. These preparations containing variable amounts of lipids, mainly dipalmitoylphosphatidylcholine (DPPC), and residual hydrophobic surfactant proteins (SP)-B and SP-C, are very effective, but supplies are limited. Development and introduction of new generation synthetic surfactants is a promising approach to further improve surfactant replacement therapy and to widen indications [8,13–17]. In contrast to natural surfactants, synthetic surfactants may have standardized composition, increased resistance against inactivation, and may avoid the need for animal reservoir. CHF5633 is a completely synthetic surfactant preparation currently being subject to clinical trials [8]. It contains a 1:1 mixture (98.3%) of DPPC and palmitoyloleylphosphatidylglycerol (POPG), a specific phosphatidylglycerol, in combination with synthetic peptide analogs to SP-B (0.2%) and SP-C (1.5%). The SP-B analog is a 34-residue peptide composed of the N- and C-terminal helical regions of surfactant protein B, but with the methionines substituted with leucines [18–20]. The SP-C analog is a 33-amino acid peptide similar to native SP-C but the N-terminal portion is truncated and contains serine instead of palmitoylated cysteines, the valines and the methionine in the middle and C-terminal regions are substituted with leucines and the leucine in position 12 is substituted with a lysine [15,20]. CHF5633 has recently been shown to be equally effective as standard, animal-derived surfactant preparations in the treatment of RDS in a preterm lamb model [15] and experimentally induced meconium aspiration syndrome in newborn pigs [21]. Moreover, in preterm lambs, superior resistance against inactivation has been found compared to the natural surfactant poractant alfa (Curosurf®) [20].

Improved survival from RDS raises the issue of longer-term lung injury and lung morbidity in preterm infants, such as bronchopulmonary dysplasia (BPD) [22–27]. Beside alveolar collapse and impaired oxygenation due to primary surfactant deficiency, RDS is characterized by a varying degree of lung inflammation [28]. Pre- and postnatal injurious events, such as chorioamnionitis, pulmonary or systemic infection, mechanical ventilation, hyperoxia and hypoxia-ischemia, have been shown to induce, aggravate and perpetuate adverse pulmonary inflammation in the structurally and immunologically immature lungs of preterm infants [23,26,28–33]. Macrophage-derived TNF-α seems to contribute significantly to this inflammatory reaction [23]. Moreover, IL-1β is a central pro-inflammatory cytokine found in the amniotic fluid in chorioamnionitis [34], that has been shown to significantly disturb lung morphogenesis in a fetal mouse model [35,36]. Increased levels of TNF-α and IL-1β were detected in tracheal aspirates of preterm infants with RDS and BPD [37]. Recruitment of inflammatory cells is mainly promoted by chemokines, thus playing a central role in regulating inflammation [38]. IL-8 (CXCL8) may be the most important chemotactic factor for recruitment of monocytes and neutrophils to the site of pulmonary inflammation [39,40]. IL-10 is a well-known anti-inflammatory cytokine inhibiting the release of pro-inflammatory mediators from monocytes and macrophages and enhancing the release of anti-inflammatory mediators [41–44]. It has been ascribed physiological relevance in prevention and limitation of adverse and injurious inflammatory immune reactions [43,45]. In the context of Gram-positive and Gram-negative infection, inflammatory cytokine release is triggered via pattern recognition receptors, such as Toll-like receptors (TLR) 2 and 4 [46]. Both TLRs have been implicated in inflammatory diseases including the pathogenesis of BPD [47–50]. Immaturity and dysregulation of TLR signaling has been hypothesized to contribute to adversely pronounced inflammatory responses in neonatal immune cells [51–53].

For natural surfactant preparations as well as some surfactant phospholipids, beneficial anti-inflammatory effects, such as surfactant-induced modulation of phagocyte function, modulation of oxidative burst, and suppression of pro-inflammatory cytokine release have been demonstrated in a relevant number of studies [54–70]. Data on potential immunomodulatory capacities of CHF5633 is limited, so far.

In the present study, we investigated the effects CHF5633 and its components on pro- and anti-inflammatory cytokine synthesis as well as TLR2 and TLR4 expression in (i) unstimulated (native) and (ii) LPS-stimulated adult human CD14^+^ monocytes.

## Material and Methods

### 2.1. Surfactant preparations

The reconstituted synthetic surfactant CHF5633 and the derived synthetic phospholipid components such as POPG, a mixture of DPPC and POPG (1:1 w/w) (PLs), PLs and SP-B analog (0.2%) (PLs+SP-B), PLs and SP-C analog (1.5%) (PLs+SP-C) as well as Curosurf® were supplied by Chiesi Farmaceutici S.p.A. (Parma, Italy).

### 2.2. Reagents

LPS from *Escherichia coli* serotype 055:B5, Polymyxin B, Brefeldin A, RPMI1640, Dulbecco’s Phosphate Buffered Saline (PBS), nuclease-free H_2_O and methanol for permeabilization were purchased from Sigma-Aldrich (St. Louis, CA). Human serum (HS) was purchased from Biochrom GmbH (Berlin, Germany) and fetal bovine serum (FBS) from Gibco Life technologies (Darmstadt, Germany). Fixation buffer containing 4% paraformaldehyde is a product of BioLegend (San Diego, CA).

### 2.3. Antibodies

Antibodies to the surface epitopes CD14 (clone HCD14, Pacific Blue-conjugated; and clone M5E2, Brilliant Violet 510-conjugated), CD16 (clone 3G8, PE-conjugated), TLR2 (clone TL2.1, PE-conjugated) and TLR4 (clone HTA125, Brilliant Violet 421-conjugated) as well as isotype controls (clone MOPC-173, clone MOPC-21, clone RTK2071) were purchased from BioLegend. For intracellular cytokine staining, antibodies to TNF-α (clone MAb11, PerCP/Cy5-5-conjugated), IL-1β (clone H1b-98, Alexa Fluor 647-conjugated), IL-8 (clone E8N1, Alexa Fluor 488-conjugated) and IL-10 (clone JES3-9D7, PE/Cy7-conjugated) were also obtained from BioLegend. Fixable viability stain (eFluor® 780, APC-H7-conjugated) was purchased from eBioScience (San Diego, CA).

### 2.4. Enrichment of adult CD14^+^ monocytes from peripheral blood mononuclear cells

Primary cells were isolated from randomized leukocyte concentrates (buffy coats) obtained from apheresis products from healthy adult donors at the Department of Immunohematology and Transfusion Medicine, University of Würzburg (http://www.transfusionsmedizin.ukw.de)–as described previously (doi: 10.1016/j.imlet.2015.05.003 [71]). Due to randomization and complete anonymization of the leukocyte concentrates, donor´s individual informed consent was not required. The study has been approved by the Ethic Committee of the Medical Faculty of Würzburg. Peripheral blood mononuclear cells (PBMCs) were accumulated from the heparinized blood on Ficoll-Paque (LINARIS Biologische Produkte GmbH, Dossenheim, Germany) for 25 min at 530×g. Adult CD14^+^ monocytes were further enriched by magnetic-activated cell sorting (MACS) using CD14 MicroBeads® (Miltenyi Biotec GmbH, Bergisch Gladbach, Germany), the corresponding MidiMACS™ separator and LS type columns (Miltenyi Biotec) according to the manufacturers´ instructions.

### 2.5. Cell culture and cell activation

CD14^+^ monocytes were re-suspended in RPMI1640 containing additional 10% FBS. For stimulation assays, cells were transferred to 24-well culture plates (Greiner, Frickenhausen, Germany) at 1×10^6^ cells/ml resting for 2h. Cells were either left unstimulated or stimulated with 100 ng/mL LPS and then exposed to 100μg/mL CHF5633, 100 μg/mL of one of the listed surfactant phospholipid preparations or 100μg/mL Curosurf®. CD14^+^ monocytes were incubated for 14 hours at 37°C in a humidified atmosphere with 5% CO_2_. Cells without LPS-stimulation and cells without exposure to pulmonary surfactants served as negative controls. In preliminary dose-response experiments, different concentrations of LPS (1 ng/ml, 10ng/ml, 100ng/ml and 1 μg/ml), CHF5633 (100μg/ml to 1mg/ml), its components (100μg/ml to 1mg/ml) and Curosurf^®^ (100μg/ml to 1mg/ml) were tested according to previous data from our group as well as other publications [54,60,64,72–74] (S1 Fig). LPS exhibited a dose-dependent induction of TNF-α, IL-1β and IL-8 with maximum expression at 100 ng/ml at qPCR (S2 Fig, data shown for TNF-α mRNA expression) and flow cytometry assessment (S3 Fig). 100ng/ml LPS did not adversely affect cell viability as confirmed by viability staining (S2B Fig). Both CHF5633 and Curosurf® showed optimal impact on LPS-induced cytokine expression at concentrations of 100μg/ml. With regard to differential kinetics of the analyzed cytokines, different incubation periods (2h, 4h, 8h, 14h, 40h) were evaluated. Flow cytometry analysis confirmed cell viability ≥ 95% in all CD14^+^ cell samples (unstimulated and stimulated) incubated for 14h and less. We verified suppresion of LPS-induced cytokine expression by treatment with 10μg/ml Polymyxin B. For intracellular cytokine flow cytometry, 10μg/ml Brefeldin A was added in order to promote the accumulation of *de novo* synthesized cytokines in the Golgi apparatus.

### 2.6. RNA extraction and Reverse transcription (RT-) PCR

For RNA extraction, CD14^+^ monocytes–treated as indicated—were harvested after 4h and 14h incubation, respectively. Cells were separated by centrifugation at 340 × g for 5 min discarding the supernatant. Total RNA was extracted using NucleoSpin® RNA II Kit (Macherey-Nagel, Düren, Germany) according to the manufacturer´s protocol. For total RNA quantitation, Qubit® 2.0 Fluorometer (Invitrogen, Life technologies) was used. Total RNA was eluted in 60μl nuclease-free water and stored at -80°C until reverse transcription. For RT-PCR, 0.27 to 0.52 μg of total RNA was reverse transcribed using High Capacity cDNA Reverse Transcription Kit (Applied Biosystems, Life Technologies, Carlsbad, CA) according to the manufacturer´s instructions. The reaction was terminated by heating at 70°C for 10 min. First strand cDNA was stored at -80° until further processing.

### 2.7. Quantitative real time PCR (qPCR)

Prior to qPCR analysis, we evaluated three frequently used housekeeping genes (peptidyl prolyl isomerase A (PPIA), PPIB and β2-microglobulin (β2M)) for analysis in native and LPS-activated adult human CD14^+^ monocytes and identified PPIA as suitable reference gene (Data not shown). For quantitative detection of TNF-α, IL-1β, IL-8, IL-10, TLR2, TLR4 and PPIA mRNA, cDNA was diluted 1:10 in deionized, nuclease-free H_2_O (Sigma) and further analyzed in duplicates of 25μl using 12.5 μL iTaq™ Universal SYBR Green Supermix (Bio-Rad Laboratories, Hercules, CA), 0.5 μL deionized H_2_O, and 1 μL of a 10 μM solution of forward and reverse primers as indicated in Table 1. Analysis was performed using a 2-step PCR protocol with 40 cycles of 95°C for 15 s and 60°C for 1 min following initial denaturing of DNA (Applied Biosystems® 7500 Real-Time PCR System, Life Technologies). A melt curve analysis was performed at the end of every run to verify single PCR products. TNF-α, IL-1β, IL-8, IL-10, TLR2 and TLR4 mRNA amplification was normalized to the reference gene PPIA (ΔCt). Mean fold changes in mRNA expression were calculated by the ΔΔC_T_ method by Livak and Schmittgen [75].

**Table 1:** Summary of the primer sequences used for qPCR and the expected product sizes in base pairs (bp).

Gene symbol	Sequence accession #	Orientation	Sequence [5´to 3´]	Amplicon length [bp]
IL-1β	NM_000576	forward	TTCATTGCTCAAGTGTCTG	128
		reverse	GCACTTCATCTGTTTAGGG	
IL-8	NM_000584	forward	CAGTGCATAAAGACATACTCC	198
		reverse	TTTATGAATTCTCAGCCCTC	
IL-10	NM_000572	forward	GCTGTCATCGATTTCTTCC	112
		reverse	GTCAAACTCACTCATGGCT	
PPIA	NM_021130	forward	CAGGGTTTATGTGTCAGGG	198
		reverse	CCATCCAACCACTCAGTC	
TLR2	NM_003264	forward	CCAAAGGAGACCTATAGTGAC	116
		reverse	GCTTCAACCCACAACTACC	
TLR4	NM_138554	forward	TTATCCAGGTGTGAAATCCA	159
		reverse	GATTTGTCTCCACAGCCA	
TNF-α	NM_000594	forward	CAGCCTCTTCTCCTTCCT	188
		reverse	GGGTTTGCTACAACATGG	

The housekeeping gene peptidyl prolyl isomerase A (PPIA) was used as reference. Normalization was performed according to the ΔΔC_T_ method by Livak and Schmittgen [75].

### 2.8. Intracellular cytokine flow cytometry

To stain intracellularly accumulated cytokines, a permeabilization strategy using ice-cold methanol was applied, as described previously by our group [71]. Adult CD14^+^ monocytes were harvested after 14h incubation and transferred into 96-well plates. Cells were separated by centrifugation at 340 × g for 5 min discarding the supernatant, and stained with antibodies against surface markers (CD14, CD16, TLR2, TLR4) and fixable viability dye for 25 min at room temperature (RT) in the dark. CD14^+^ cells were separated by centrifugation again, washed in PBS containing 1% HS, and fixed for 30 min using fixation buffer. Surface-stained and fixed CD14^+^ monocytes were centrifuged at 340 × g for 5 min, re-suspended in ice-cold methanol, and incubated for 30 min on ice in the dark. Cells were washed twice with PBS/1% HS and stained with directly conjugated anti-cytokine antibodies (TNF-α, IL-1β, IL-8 and IL-10), that had been dissolved in PBS/1% HS, for 45 min at RT in the dark. Cells were washed once in PBS/1% HS and finally re-suspended in PBS/1% HS.

### 2.9. Flow cytometry analysis

All specimens were analyzed using a FACSCanto™ II flow cytometer (BD Biosciences, Franklin Lakes, NJ). Instrument set-up and compensation/calibration were performed prior to data acquisition. A baseline fluorescence control and an isotype-matched negative control were used as a reference to set the fluorescence thresholds for positivity. Results for cytokine positive cells (mean ± SD) are expressed as the percentage of the respective subpopulation. A minimum of 10.000 CD14^+^ monocyte-gated events was acquired in list mode and analyzed with FACSDiva v6.1.3 software (BD Biosciences). Events were gated on monocytes via forward and side scatter and for CD14^+^ Viability-dye^-^ cells (S4 Fig). According to Herzenberg *et al*. [76], fluorescence minus one (FMO) was used to set the marker for positive cells. Results are presented as percentage of CD14^+^ monocytes positive for TNF-α, IL-1β, IL-8 and IL-10 as well as TLR2 and TLR4. For measurement of the spectral overlaps, the fluorescence detected on all measurement channels was evaluated for single-labeled “compensation control” samples prior to the performed FACS analysis.

### 2.10. Statistical analysis

All results shown are combinations of a minimum of four independent experiments. Results are given as means ± standard deviation (SD). Data were analyzed using the nonparametric Kruskal-Wallis test with Dunn’s multiple comparison post hoc test. A p-value ≤ 0.05 was considered significant. Statistical differences were denoted as follows: * = p < 0.05; ** = p < 0.01; *** = p < 0.001. Statistical analyses were performed using Prism® version 6 (GraphPad Software, San Diego, CA).

## Results

### Cell culture characteristics

Bead-selected and cultured cells were > 92% CD14^+^ monocytes as determined by flow cytometry analysis. The majority of CD14^+^ monocytes (> 95%) were CD14^+^CD16^-^ cells. Cell viability was confirmed ≥ 95% by viability staining and was shown to be unaffected by 4h and 14h cell culture as well as 4h and 14h treatment with LPS. Viability staining revealed increasing apoptosis in unstimulated CD14^+^ cells cultured for 40h and more. Thus, incubation with the given surfactant preparations was limited to 14h in all given experiments.

### Basal and LPS-induced cytokine expression

**Fig 1:**
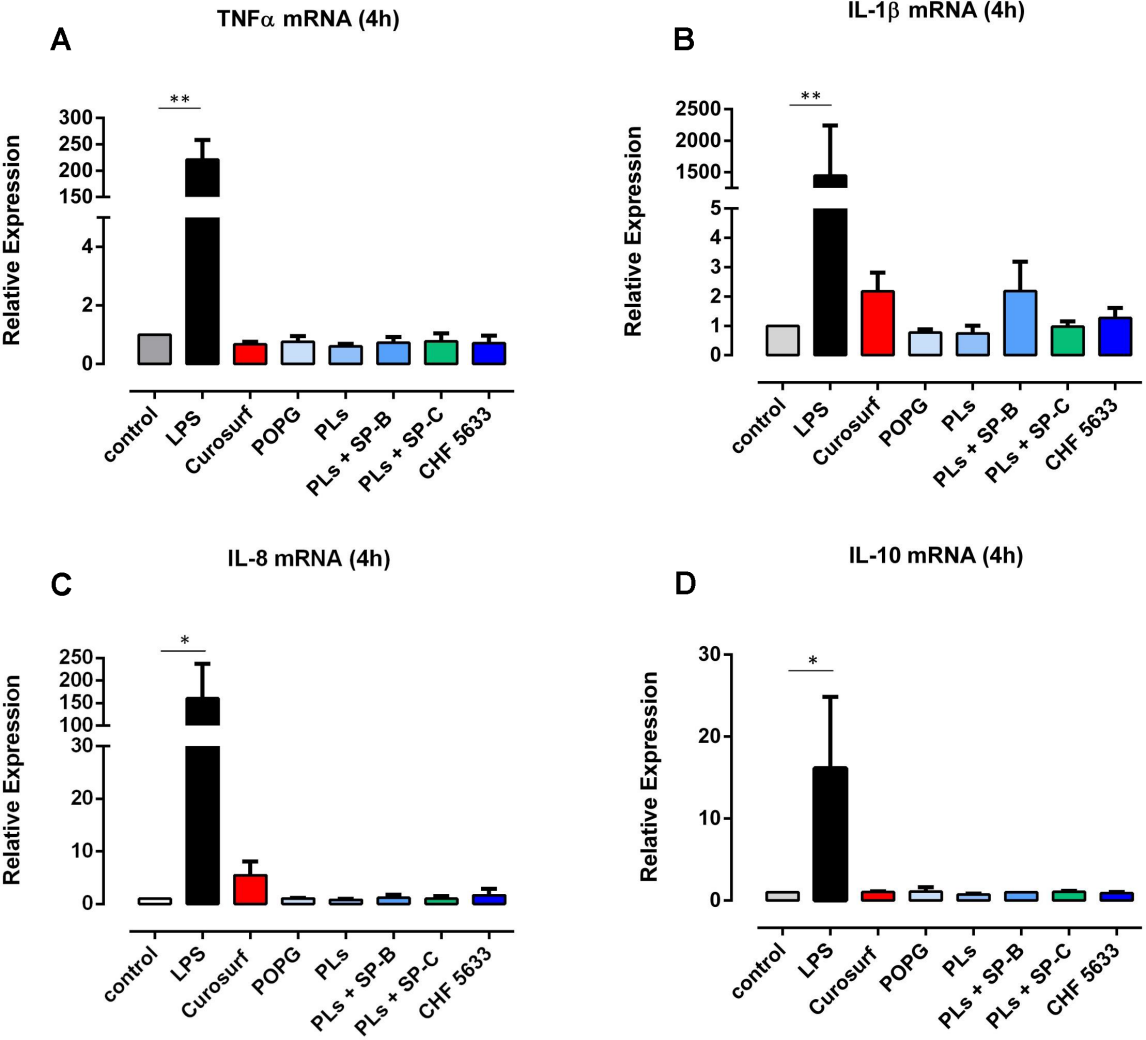
Pro- and anti-inflammatory cytokine mRNA expression in native CD14^+^ monocytes. Unstimulated adult CD14^+^ monocytes were exposed to 100μg/ml Curosurf, 100μg/ml POPG, 100μg/ml PLs, 100μg/ml PLs+SP-B, 100μg/ml PLs+SP-C and 100μg/ml CHF5633 for 4h. Cells were lysed and total RNA was extracted for TNF-α (A), IL-1β (B), IL-8 (C) and IL-10 mRNA (D) quantification by real-time quantitative PCR. LPS-stimulated (100ng/ml) CD14^+^ cells served as positive control. Relative expression is given as mean ± SD of 4 independent experiments (*p < 0.05, **p < 0.01).

**Fig 2:**
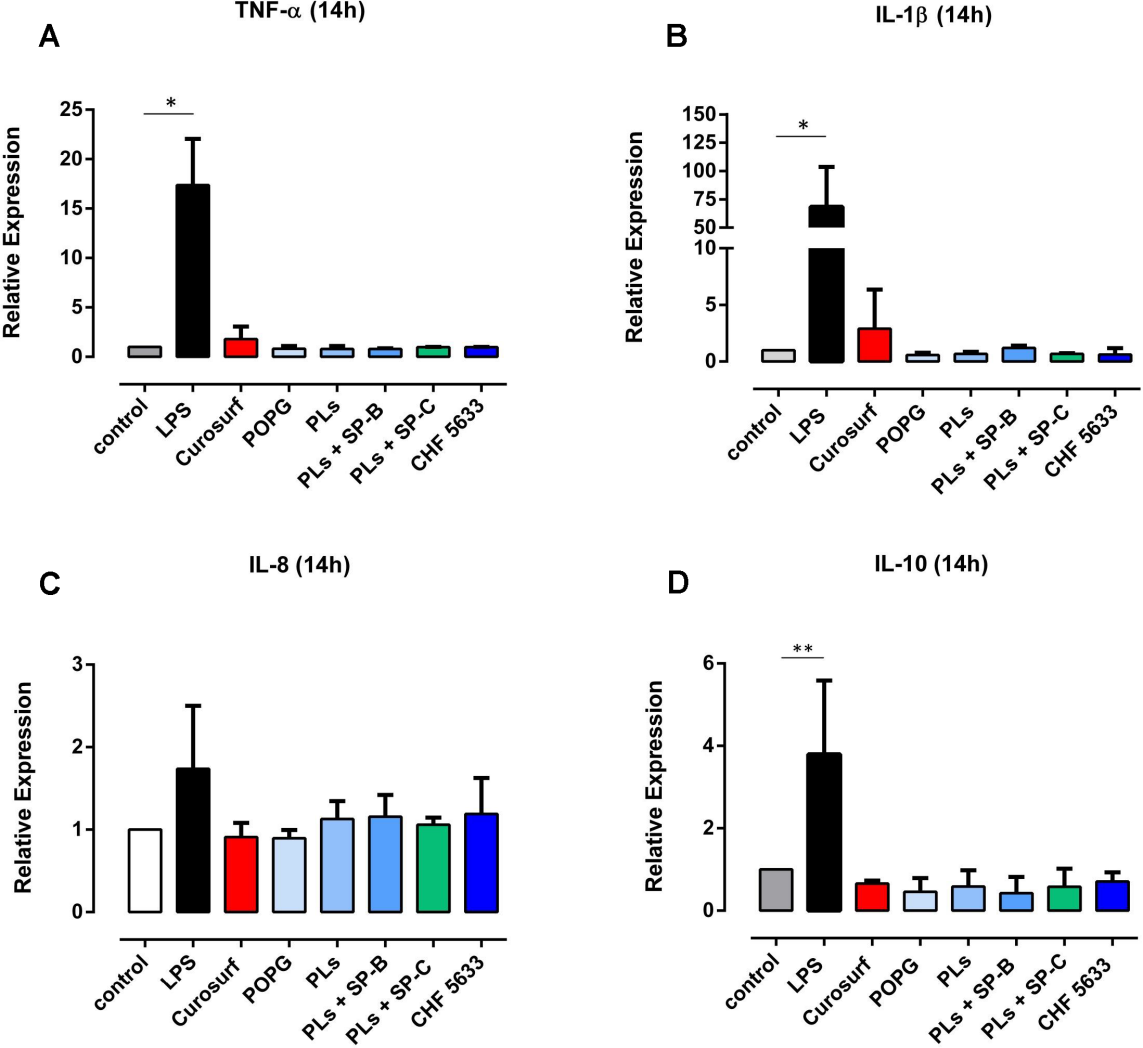
Intracellular synthesis of pro- and anti-inflammatory cytokines in native CD14^+^ monocytes. Native CD14^+^ cells (n = 4) were incubated with LPS 100ng/ml, 100μg/ml Curosurf, 100μg/ml POPG, 100μg/ml PLs, 100μg/ml PLs+SP-B, 100μg/ml PLs+SP-C and 100μg/ml CHF5633 for 14h. Unstimulated CD14^+^ cells served as negative control, LPS-stimulated monocytes served as positive control. Intracellular concentrations of TNF-α (A), IL-1β (B), IL-8 (C) and IL-10 protein (D) are given as mean ± SD (*p < 0.05, **p < 0.01).

**Fig 3:**
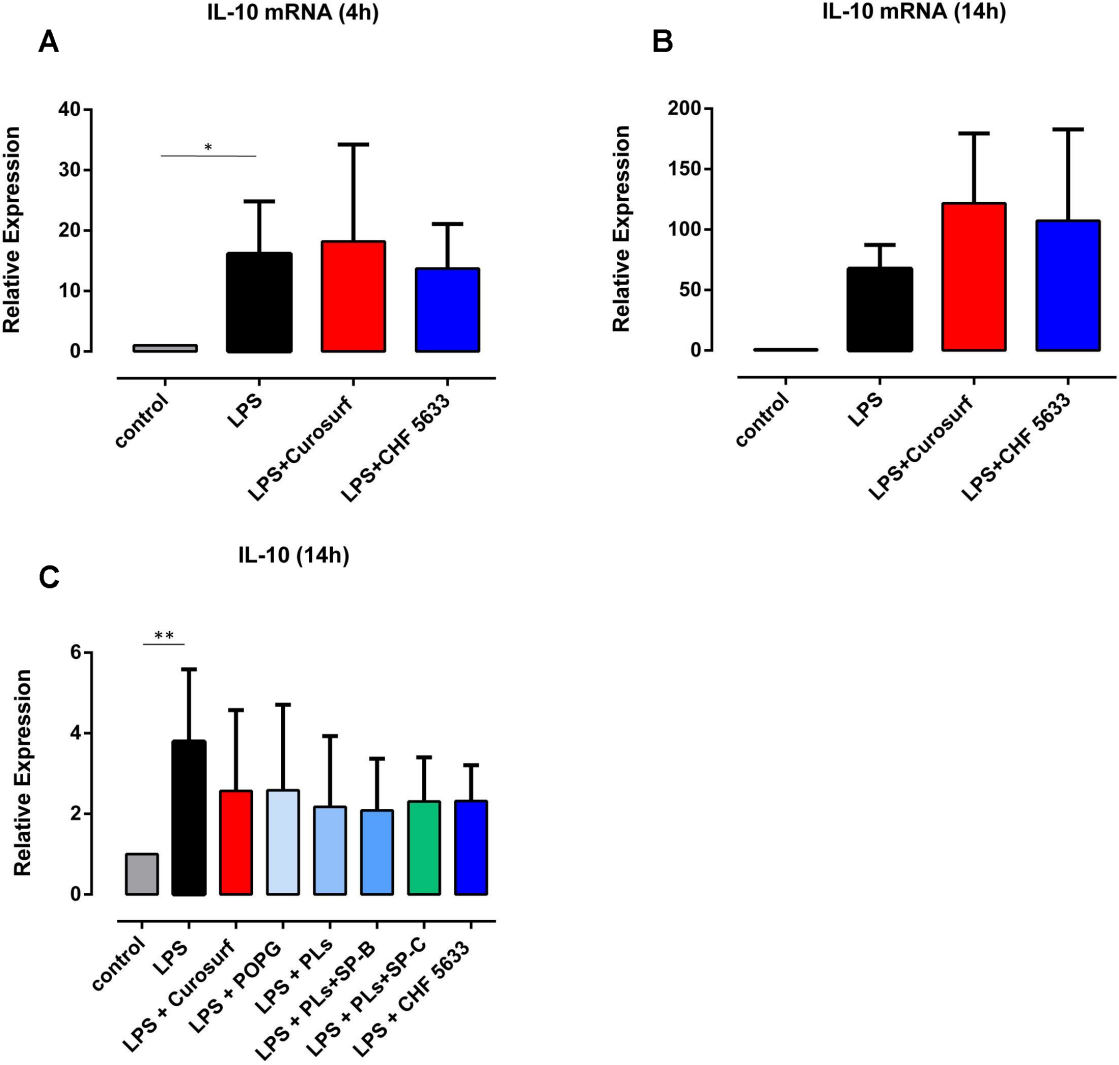
Effects of CHF5633 on IL10 mRNA and protein expression in LPS-activated adult human monocytes. Monocytes were stimulated with 100ng/ml LPS and subsequently exposed to 100μg/ml CHF5633 or 100μg/ml Curosurf®(A, B) or 100μg/ml POPG, 100μg/ml PLs, 100μg/ml PLs+SP-B and 100μg/ml PLs+SP-C, respectively (C). IL-10 mRNA expression was assessed at 4h (A) and 14h (B) qPCR. IL-10 protein expression was analyzed by means of flow cytometry at 14h (C). Results are expressed as mean ± SD (*p < 0.05, **p < 0.01).

Pro-inflammatory TNF-α, IL-1β and IL-8 mRNA expression in native (non-activated) adult human monocytes was quantified by real-time quantitative PCR. Native adult monocytes displayed hardly any TNF-α, IL-1β and IL-8 mRNA expression at 4h and 14h assessment (Fig 1, data shown for 4h qPCR analysis). On the contrary, 4h and 14h incubation of adult CD14^+^ monocytes with 100ng/ml *E coli*. LPS resulted in a significant increase in TNF-α, IL-1β and IL-8 mRNA expression levels (4h: TNF-α mean 221 ± 37, p = 0.0013, IL-1β mean 1448 ± 593, p = 0.0028, IL-8 mean 161 ± 69, p = 0.038; 14h: TNF-α mean 2.73 ± 0.33, p = 0.033, IL-1β mean 2.21 ± 0.40, p = 0.031, IL-8 mean 2.14 ± 0.37, p = 0.039; compared to unstimulated controls) (Fig 1, 14h data not shown). Flow cytometry analysis revealed significantly increased synthesis of intracellular TNF-α and IL-1β protein in LPS-stimulated CD14^+^ monocytes compared to unstimulated controls (TNF-α mean 17.4 ± 4.6, p = 0.020; IL-1β mean 68.4 ± 35.2, p = 0.031) (Fig 2A and 2B). As far as IL-8 protein expression was concerned, LPS-induced increase was not significant (mean 1.74 ± 0.76, p = 0.14) (Fig 2C). TNF-α, IL-1β and IL-8 mRNA expression levels continuously decreased at 8h and 14h analysis (data not shown). Native CD14^+^ monocytes showed negligible anti-inflammatory IL10 mRNA and protein expression (Figs 1D and 2D). Even upon LPS stimulation, IL-10 mRNA and protein expression levels were lower than pro-inflammatory cytokine levels (Fig 3) (4h mRNA: mean 16.2 ± 8.7, p = 0.042; 14h mRNA: mean 59.4 ± 27.3, p = 0.18; 14h protein: mean 3.76 ± 1.66, p = 0.005, vs. unstimulated controls).

### Effects of CHF5633 and its components on cell viability and pro- and anti-inflammatory cytokine expression in native adult human CD14^+^ monocytes

**Fig 4:**
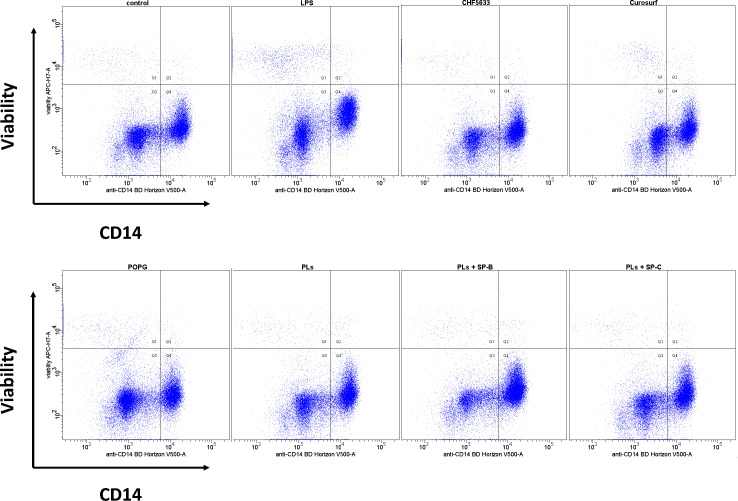
Flow cytometry analysis of cell death in CD14^+^ monocytes after exposure of cells to LPS and surfactant preparations. Viability of cells was assessed by flow cytometry staining using APC-H7-conjugated viability dye labeling dead cells prior to staining for intracellular antigens. Dead cells are displayed in upper left and upper right quadrant.

Viability of adult CD14^+^ monocytes was unaffected by exposure of cells to 100μg/ml CHF5633, its components and Curosurf® as assessed by flow cytometry (Fig 4). Moreover, incubation of adult monocytes with 100μg/ml CHF5633 did not increase TNF-α, IL-1β and IL-8 mRNA expression, neither at 4h nor at 14h incubation time (Fig 1A–1C, 4h data given). Identical results were obtained for POPG, PLs, PLs+SP-B and PLs+SP-C (Fig 1A–1C). These findings are in accordance with negligible monocytic TNF-α, IL-1β and IL-8 mRNA expression after exposure to Curosurf® for 4h and 14h, respectively. In agreement to qPCR results, neither CHF5633 nor its components, nor Curosurf® significantly induced intracellular TNF-α, IL-1β and IL-8 protein synthesis in native adult human monocytes (Fig 2A–2C). Anti-inflammatory IL10 mRNA and protein expression were also unaffected by incubation of CD14^+^ cells with CHF5633, its components or Curosurf® (Figs 1D and 2D).

### Differential effects of CHF5633 on LPS-induced cytokine expression in adult CD14^+^ cells

**Fig 5:**
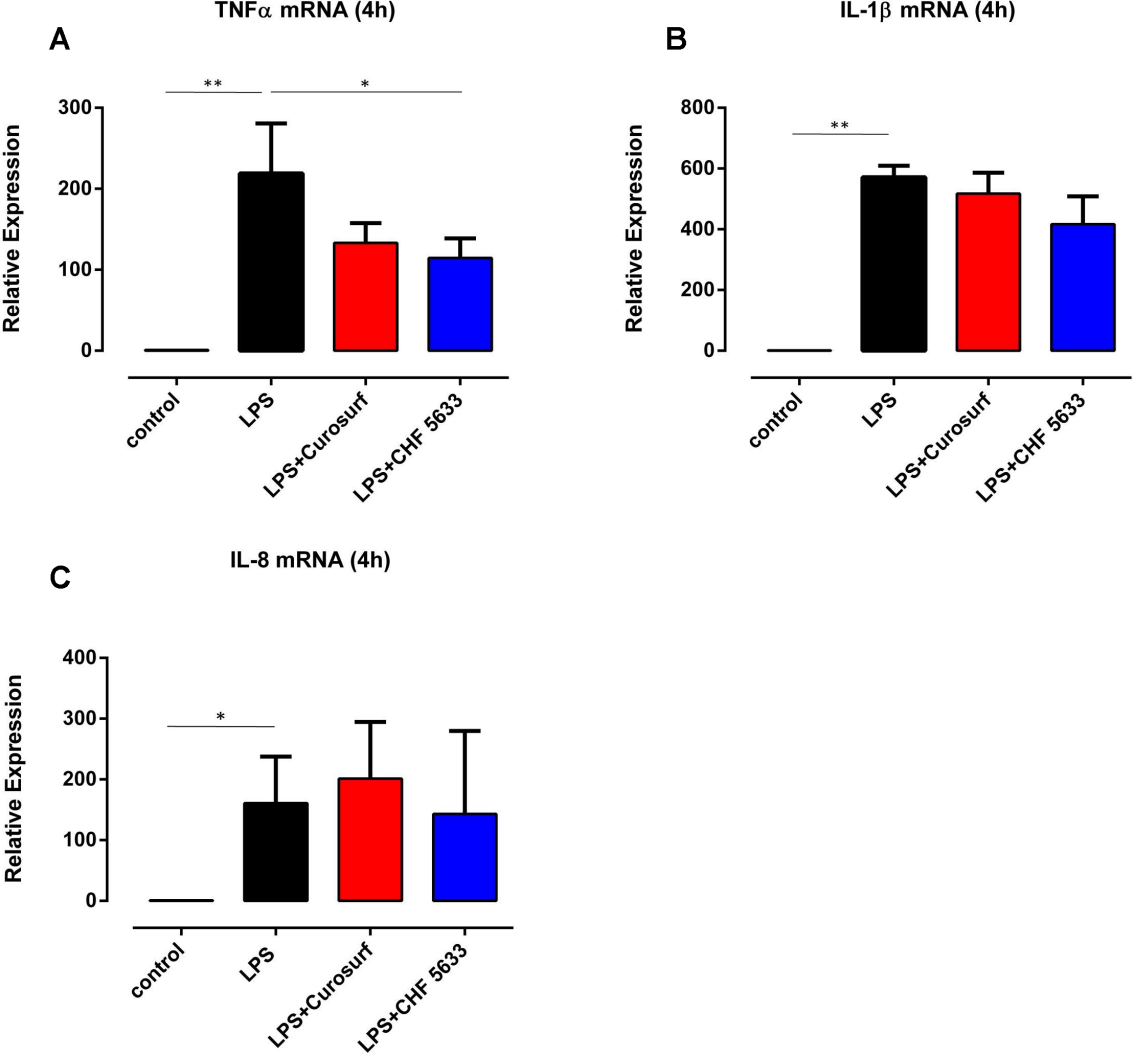
Effects of CHF5633 on LPS-induced pro-inflammatory cytokine mRNA expression in adult CD14^+^ monocytes. TNF-α (A), IL-1β (B) and IL-8 (C) mRNA expression were assessed in LPS-stimulated adult CD14^+^monocytes (n = 4) simultaneously exposed (4h) to 100μg/ml CHF5633 or 100μg/ml Curosurf®. Unstimulated CD14^+^ cells served as negative control, LPS-stimulated monocytes (100ng/ml) as positive control. Results are expressed as mean ± SD (*p < 0.05; **p < 0.01).

**Fig 6:**
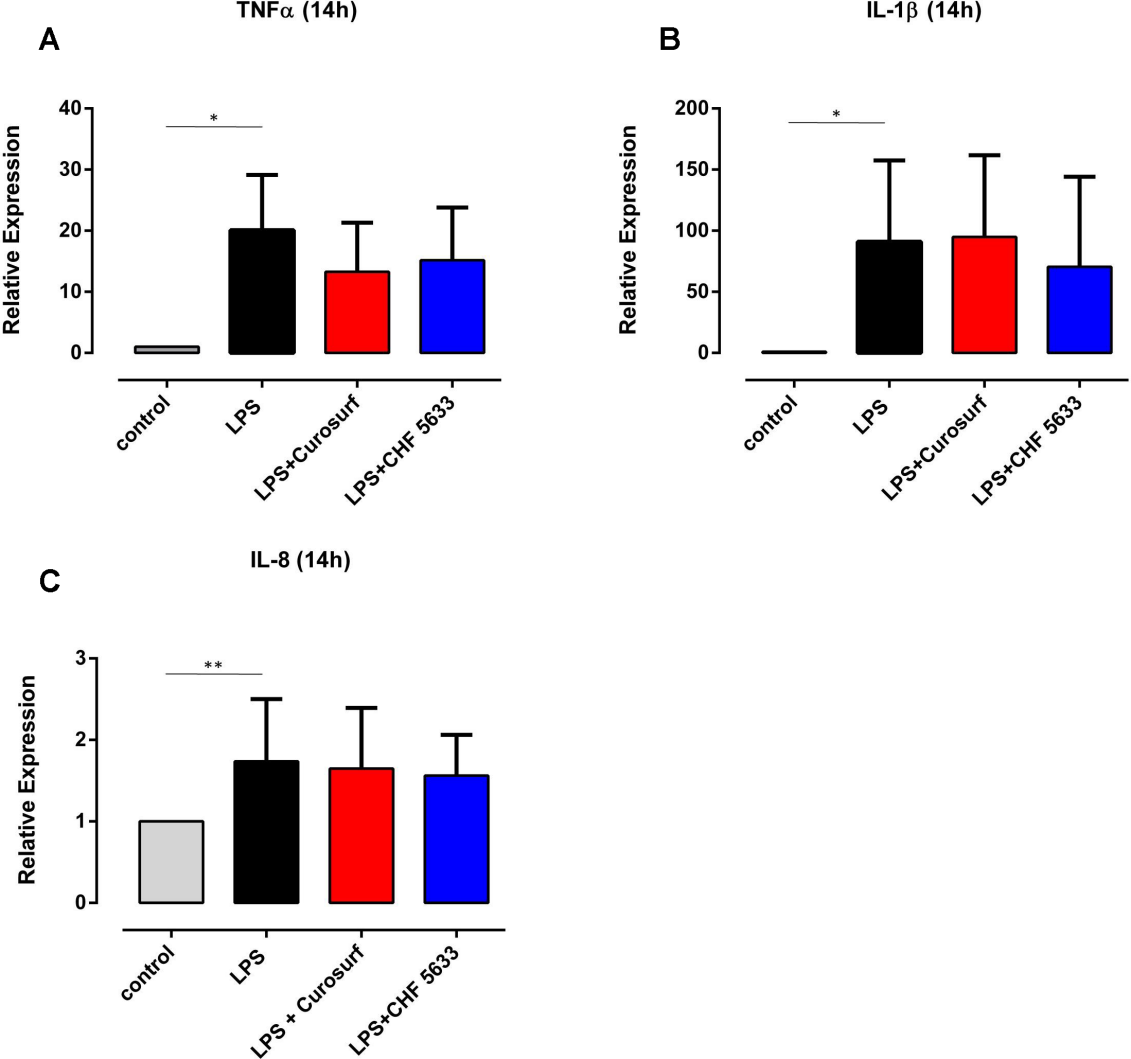
Effects of CHF5633 on LPS-induced intracellular pro-inflammatory cytokine synthesis in adult CD14^+^ monocytes. The diagrams illustrate relative expression of intracellular TNF-α (A), IL-1β (B) and IL-8 protein (C) in LPS-stimulated CD14^+^ cells after 14h additional exposure to 100μg/ml CHF5633 or 100μg/ml Curosurf®. Non-exposed CD14^+^ cells served as negative, LPS-activated monocytes as positive controls. Results (n = 4) are expressed as mean ± SD (*p < 0.05, **p < 0.01).

Simultaneous exposure of LPS-stimulated adult monocytes to 100μg/ml CHF5633 led to a significant reduction of TNF-α mRNA expression at 4h assessment relative to monocytes exposed only to LPS (0.57 ± 0.23-fold, p = 0.043) (Fig 5A). After 14h incubation, CHF5633-exposed LPS-activated monocytes showed TNF-α mRNA expression levels that were >50% lower than levels found in LPS-activated controls (0.56 ± 0.27-fold, p = 0.042) (data not shown). 4h exposure of LPS-stimulated adult monocytes to Curosurf® led to a similar, but not statistically significant reduction of LPS-induced TNF-α mRNA expression of almost 40% (0.63 ± 0.13-fold, p = 0.29) (Fig 5A). Moreover, for none of the tested components of CHF5633, a significant reduction of TNF-α mRNA in LPS-stimulated monocytes was observed (S5A Fig). As far as IL-1β was concerned, a slight, but not significant reduction of mRNA expression was found following monocyte exposure to CHF5633 (0.73 ± 0.16-fold, p = 0.17) (Fig 5B). Curosurf®-exposure did not affect LPS-induced IL-1β mRNA expression (Fig 5B), neither did exposure of cells to one of the tested components of CHF5633 (S5B Fig). LPS-induced IL-8 mRNA expression was unaffected by CHF5633, its components, and Curosurf® (Fig 5C, S5C Fig). Assessing intracellular cytokines by means of flow cytometry, we found a tendency towards reduced TNF-α protein synthesis in adult monocytes that had been exposed either to CHF5633 or Curosurf® (Fig 6A). However, these results did not reach statistical significance. As far as intracellular IL-1β and IL-8 were concerned, neither exposure of cells to CHF5633, nor exposure to Curosurf® affected protein expression (Fig 6B and 6C). Moreover, incubation of LPS-activated adult monocytes with CHF5633´s components PLs, PLs+SP-B and PLs+SP-C did not affect IL-1β and IL-8 protein expression (data not shown).

Anti-inflammatory IL-10 mRNA expression did not differ significantly following LPS treatment alone and simultaneous exposure to CHF5633 at 4h assessment (Fig 3A), but was 50% higher in LPS-stimulated adult monocytes having been exposed to CHF5633 for 14h compared to LPS-activated controls. However, this finding did not reach statistical significance (mean 1.56 ± 1.10-fold, p = 0.83 vs. LPS-stimulated monocytes) (Fig 3B). Curosurf® exposure of LPS-stimulated monocytes paralleled those results (mean at 14h 1.77 ± 0.49-fold, p = 0.11 vs. LPS-stimulated monocytes). At 14h incubation, flow cytometry revealed a slight, but not significant increase in intracellular IL-10 protein synthesis in CHF5633- and Curosurf®-exposed, LPS-activated CD14^+^ monocytes compared to surfactant exposure alone (Figs 2D and 3C). However, the latter induction of IL-10 did not exceed LPS-induced IL-10 protein expression. Exposure to POPG, PLs, PLs+SP-B and PLs+SP-C did not significantly affect either IL-10 mRNA or protein expression in LPS-stimulated monocytes (Fig 3C, data shown for protein expression).

### CHF5633, its components and Curosurf^®^ did not induce TLR2 and TLR4 expression in native and LPS-activated adult CD14^+^ cells

**Fig 7:**
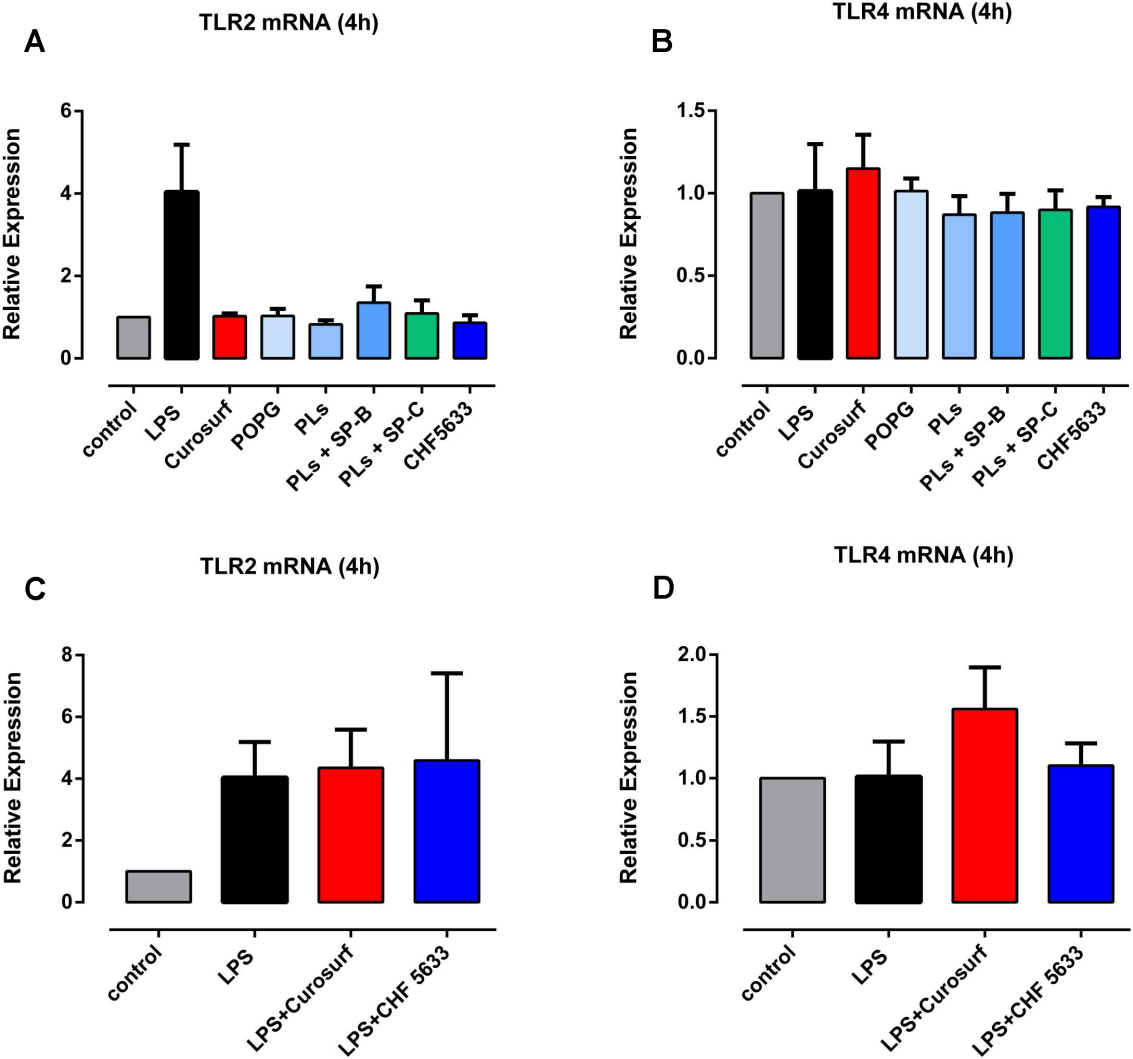
Effect of CHF5633, its derived synthetic surfactant preparations and Curosurf^®^ on TLR2 and TLR4 mRNA expression in non-activated and LPS-stimulated CD14^+^ monocytes. Relative expression of TLR2 and TLR4 mRNA in non-activated (A, B) and LPS-stimulated (C, D) CD14^+^ monocytes after 4h exposure to 100μg/ml CHF5633, 100μg/ml POPG, 100μg/ml PLs, 100μg/ml PLs+SP-B, 100μg/ml PLs+SP-C and 100μg/ml CHF5633 is given. LPS-activated CD14^+^ cells (100ng/ml) served as positive control. Relative concentrations of TLR2 and TLR4 are given as mean ± SD (n = 3).

As can be seen in Fig 7, native adult monocytes displayed limited TLR2 and TLR4 mRNA expression at 4h qPCR assessment (Fig 7A and 7B). Upon stimulation with LPS, monocytes showed a non-significant increase in TLR2 mRNA expression (Fig 7A and 7C) (mean 3.83 ± 1.03, p = 0.12) and unaltered TLR4 mRNA expression (Fig 7B). Exposure of native adult monocytes to 100μg/ml CHF5633 did not increase TLR2 and TLR4 mRNA expression at 4h incubation (Fig 7A and 7B). Identical results concerning TLR2 and TLR4 mRNA expression levels were obtained in the context of POPG, PLs, PLs+SP-B and PLs+SP-C exposure as well as Curosurf® (Fig 7A and 7B). In LPS-activated adult monocytes, CHF5633, its components and Curosurf®, again, did not affect TLR2 and TLR4 mRNA expression levels (Fig 7C and 7D). Flow cytometry analysis at 14h incubation assessed negligible amounts of TLR2 and TLR4 protein expression in native CD14^+^ monocytes. Moreover, low TLR2 and TLR4 protein expression was unaffected by both LPS stimulation and exposure of monocytes to CHF5633, its components and Curosurf® (data not shown).

## Discussion

Monocytes and alveolar macrophages are a well-known source of robust cytokine response. Our data may demonstrate that CHF5633 does not exert unintended pro-inflammatory effects on native and LPS-activated adult human monocytes. In native monocytes, pro-inflammatory TNF-α, IL-1β and IL-8 mRNA and protein expression were not increased in CHF5633-exposed cells compared to the generally low expression levels of non-exposed native controls. On the contrary, in LPS-activated adult monocytes, TNF-α mRNA expression was significantly suppressed by exposure to CHF5633, paralleled by a non-significant downward trend in intracellular TNF-α protein synthesis. For IL-1β mRNA and protein expression, similar trends did not reach statistical significance. LPS-induced IL-8 mRNA and protein expression were unaffected by CHF5633. Effects of CHF5633 on LPS-induced TNF-α and IL-1β expression paralleled those of Curosurf®. The latter results are in accordance with previous data from our group and with results from a number of other studies on natural surfactants such as Curosurf® [54,56,58,62,63,65,68–70]. Although described in previous studies [64,67,74], we did not find an equivalent anti-inflammatory activity when evaluating the major synthetic components of CHF5633 alone.

As far as anti-inflammatory IL-10 is concerned, we found a slightly, but not significantly enhanced mRNA but not protein expression in LPS-stimulated monocytes following 14h exposure to both CHF5633 and Curosurf®. Inconsistency of the presented data may be due to a relevant delay of protein synthesis as it is known for many inflammatory transcripts [44]. Severely delayed kinetics have been reported for IL-10 protein accumulation following IL-10 transcripts [77], and assessment time might have been too early. IL-10 seems to be produced rather late and after pro-inflammatory mediators [43]. Of note, CHF5633-induced enhancement of IL-10 mRNA expression at 14h exceeded CHF5633-induced enhancement at 4h, but was less pronounced than CHF5633-induced suppression of TNF-α mRNA expression at 4h relative to controls. This finding might be of relevance for homeostasis under physiological conditions, since exaggerated IL-10 expression might limit host immune response, leading to persistent inflammation [43,44].

To our knowledge, this is the first study addressing modulation of TLR2 and TLR4 expression by synthetic surfactants in human monocytes. Neither CHF5633 nor its components affected TLR2 or TLR4 mRNA and protein expression. This finding might be of relevance, since induction of TLR2 and TLR4 expression might render monocytes particularly alert for invading microorganisms.

## Conclusion

According to our present data, one may postulate that CHF5633 does not exert unintended pro-inflammatory effects. Moreover, there is some evidence, that in the context of pre-existing lung inflammation, CHF5633 might even have a limitating or reducing effect on pulmonary inflammation by decreasing TNF-α mRNA expression in human adult monocytes. These characteristics might encourage the application of CHF5633 in surfactant replacement therapy. However, conclusions are limited by the use of adult monocytes in the present study. Based on this first evaluation of potential immunomodulatory capacities of CHF5633, our future study will address pro- and anti-inflammatory features of CHF5633 in cord blood monocytes.

## Supporting Information

S1 Fig Preliminary dose-response experiments evaluating cell viability of adult CD14^+^ monocytes at 14h exposure to 100μg/ml and 1mg/ml Curosurf® as well as 100μg/ml and 1mg/ml CHF5633 (n = 3). (TIF)

S2 Fig Preliminary dose-response study with *E*. *coli* (055:B5) LPS. (A) LPS caused a dose-dependent induction of TNF-α mRNA expression in purified adult CD14^+^ monocytes at 4h qPCR assessment (n = 3), without adversely affecting cell viability (B).
(TIF)

S3 Fig Preliminary dose-response study with *E*. *coli* (055:B5) LPS. LPS induced significant expression of intracellular TNF-α, IL-1β and IL-8 at concentrations of 100ng/ml.
(TIF)

S4 Fig Gating strategy applied in this study to quantify intracellular cytokine synthesis in CD14^+^ monocytes by polychromatic flow cytometry. A representative sample of LPS-stimulated adult monocytes exposed to CHF5633 is displayed in forward and sideward scatter plot (A). Doublets were excluded by using a FSC-height versus FSC-width dot plot (B). Keeping in mind the continuous differentiation of monocytes [78], events were gated for CD14^+^ viability-dye^-^ cells (C) to maximize homogeneity and representativeness of the analyzed cell population. Contour plots identifying CD14^+^ viability-dye^-^ cytokine^+^ cell subsets are given as follows: CD14^+^ TNF-α^+^ (D), CD14^+^ IL-1β^+^ (E), CD14^+^ IL-8^+^ (F) and CD14^+^ IL-10^+^ (G). According to Herzenberg *et al*. [76], fluorescence minus one (FMO) was used to set the marker for positive cells.
(TIF)

S5 Fig Effects of CHF5633 and its synthetic phospholipid components on LPS-induced pro-inflammatory cytokine mRNA expression in adult CD14^+^ monocytes. TNF-α (A), IL-1β (B) and IL-8 (C) mRNA expression were assessed in LPS-stimulated adult CD14^+^monocytes (n = 4) simultaneously exposed to 100μg/ml CHF5633, 100μg/ml POPG, 100μg/ml PLs, 100μg/ml PLs+SP-B and 100μg/ml PLs+SP-C, or 100μg/ml Curosurf®. Unstimulated CD14^+^ cells served as negative control, LPS-stimulated monocytes (100ng/ml) as positive control. Results are expressed as mean ± SD (*p < 0.05; **p < 0.01).
(TIF)
