# Supplementary figures and images for: Effects of the New Generation Synthetic Reconstituted Surfactant CHF5633 on Pro- and Anti-Inflammatory Cytokine Expression in Native and LPS-Stimulated Adult CD14+ Monocytes
Source: PLoS One. 2016 Jan 20;11(1):e0146898. doi: 10.1371/journal.pone.0146898 (PMC4720484; doi:10.1371/journal.pone.0146898)

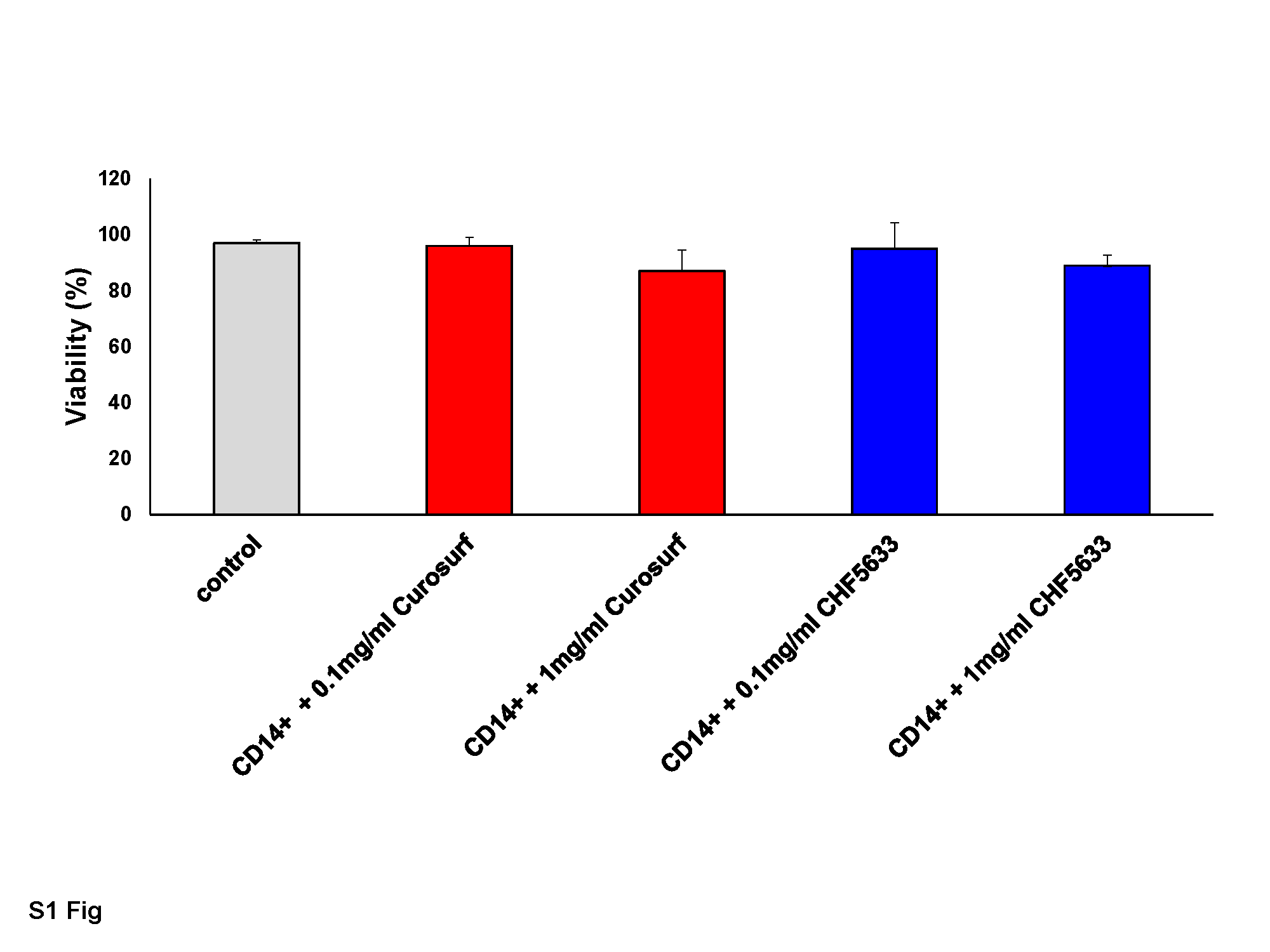

Supplement: S1 Fig — (TIF) [file pone.0146898.s001.tif]

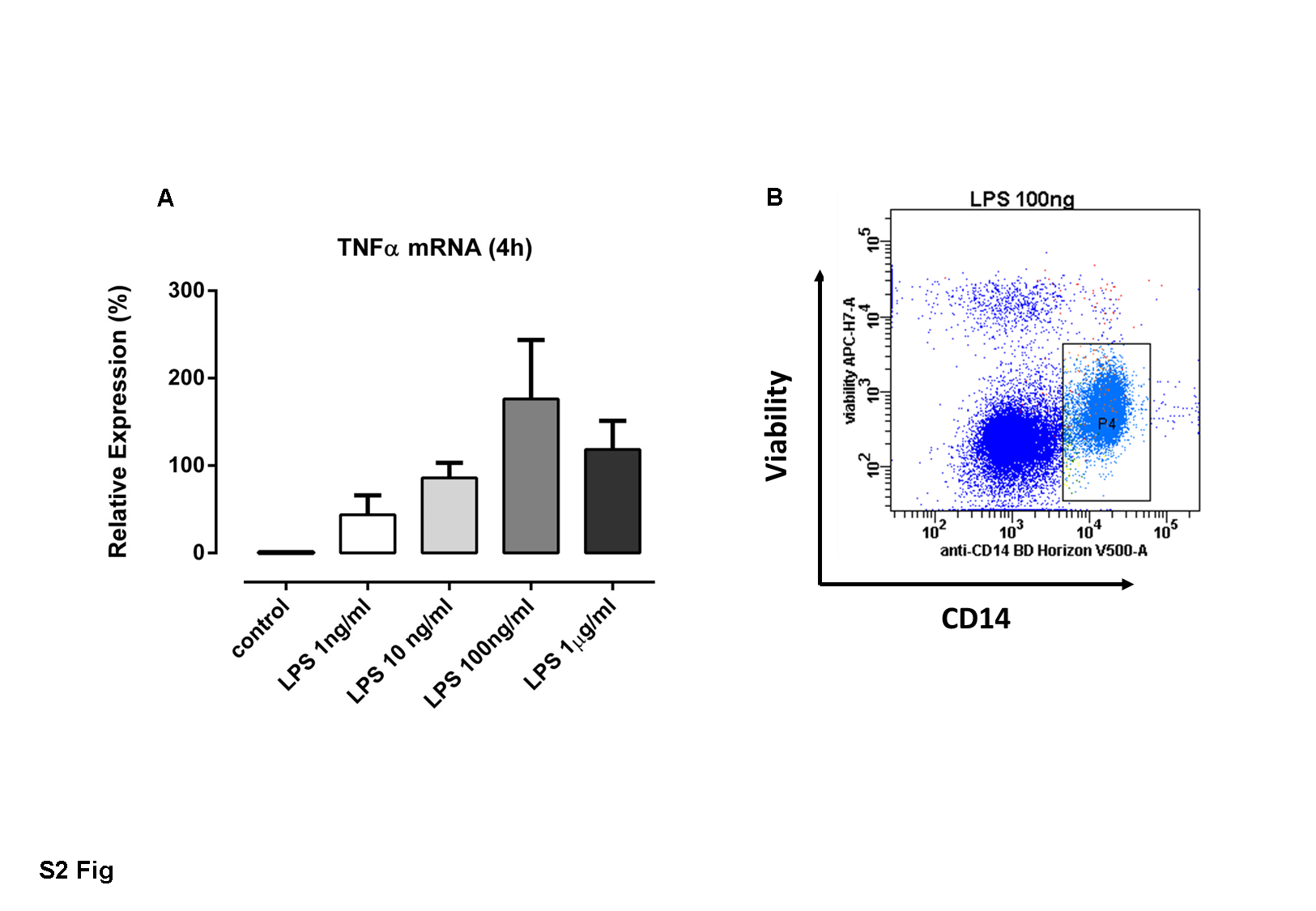

Supplement: S2 Fig — (A) LPS caused a dose-dependent induction of TNF-α mRNA expression in purified adult CD14+ monocytes at 4h qPCR assessment (n = 3), without adversely affecting cell viability (B). (TIF) [file pone.0146898.s002.tif]

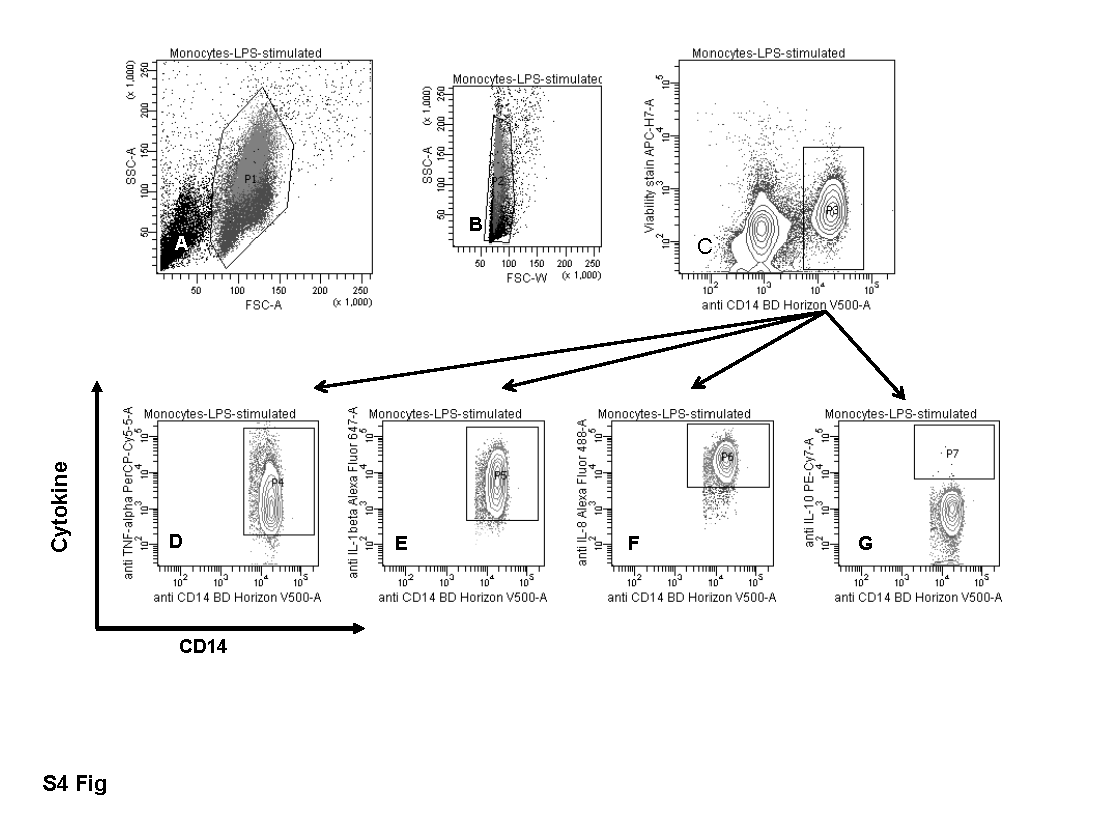

Supplement: S4 Fig — A representative sample of LPS-stimulated adult monocytes exposed to CHF5633 is displayed in forward and sideward scatter plot (A). Doublets were excluded by using a FSC-height versus FSC-width dot plot (B). Keeping in mind the continuous differentiation of monocytes [78], events were gated for CD14+ viability-dye- cells (C) to maximize homogeneity and representativeness of the analyzed cell population. Contour plots identifying CD14+ viability-dye- cytokine+ cell subsets are given as follows: CD14+ TNF-α+ (D), CD14+ IL-1β+ (E), CD14+ IL-8+ (F) and CD14+ IL-10+ (G). According to Herzenberg et al. [76], fluorescence minus one (FMO) was used to set the marker for positive cells. (TIF) [file pone.0146898.s004.tif]

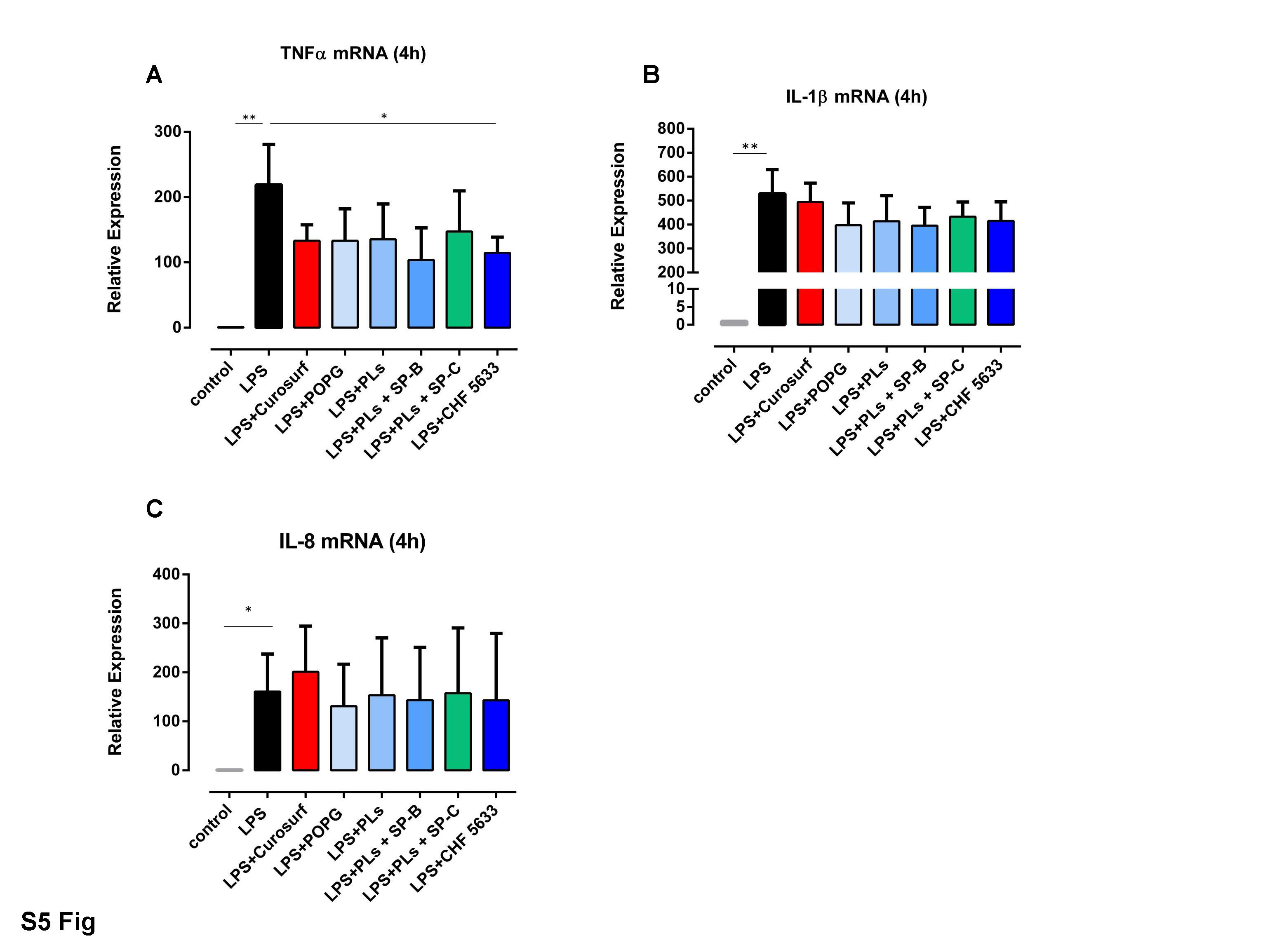

Supplement: S5 Fig — TNF-α (A), IL-1β (B) and IL-8 (C) mRNA expression were assessed in LPS-stimulated adult CD14+monocytes (n = 4) simultaneously exposed to 100μg/ml CHF5633, 100μg/ml POPG, 100μg/ml PLs, 100μg/ml PLs+SP-B and 100μg/ml PLs+SP-C, or 100μg/ml Curosurf®. Unstimulated CD14+ cells served as negative control, LPS-stimulated monocytes (100ng/ml) as positive control. Results are expressed as mean ± SD (*p < 0.05; **p < 0.01). (TIF) [file pone.0146898.s005.tif]
